# High-Pressure Treatment of Non-Hydrated Flour Affects Structural Characteristics and Hydration

**DOI:** 10.3390/foods7050078

**Published:** 2018-05-16

**Authors:** Sabina Jakobi, Mario Jekle, Thomas Becker

**Affiliations:** Research Group Cereal Technology and Process Engineering, Institute of Brewing and Beverage Technology, Technical University of Munich, 85354 Freising, Germany; sabina.jakobi@tum.de (S.J.); tb@tum.de (T.B.)

**Keywords:** starch modification, wheat flour, physical modification, starch alteration

## Abstract

In recent years, high-pressure treatment (HPT) has become an established process concerning the preservation of food. However, studies dealing with the structural, and consequently functional modification of non-hydrated starchy matrices (moisture content ≤ 15%) by HPT are missing. To close this knowledge gap, pressure (0–600 MPa, 10 min) and pressurization time depending (0–20 min, 450 MPa) alterations of wheat flour were investigated. Pressure rise from 0 to 600 MPa or pressurization time rise from 0 to 20 min resulted in a decline of amylopectin content from 68.3 ± 2.0% to 59.7 ± 1.5% (linearly, *R*^2^ = 0.83) and 59.6 ± 0.7% (sigmoidal), respectively. Thereby, detectable total amount of starch decreased from 77.7 ± 0.8% linearly to 67.6 ± 1.7%, and sigmoidal, to 69.4 ± 0.4%, respectively. Increase in pressure caused a linear decrease in gelatinization enthalpy of 33.2 ± 5.6%, and linear increase in hydration properties by 11.0 ± 0.6%. The study revealed structural and technological relevant alterations of starch-based food matrices with low moisture content by HPT, which must be taken into consideration during processing and preservation of food.

## 1. Introduction

In recent years, consumer expectations on safety, sensory, and nutritive characteristics of processed food have increased, resulting in the evolution of novel processing technologies as high-pressure treatment (HPT). Thereby in the beverage industry, HPT diminishes the microbial load of products without significantly affecting quality parameters [1,2]. However, studies performed on cereal biopolymers revealed HPT induced alterations of matrices, resulting in the usage of HPT for the functionalization and beneficial modification of product quality [3,4,5,6]. In gluten-free products, HPT of corn or rice starches resulted in a reduced staling rate of breadcrumbs due to structural alterations of starch, and thus, extended the shelf-life of the products [7]. 

Although in some food matrices, HPT should not cause alteration of covalent bonds and applied pressure should have minimal effects on processed food [8], research dealing with starchy matrices investigated great changes of polymers resulting in modified enzymatic reactions. Pressure-treated potato starch or barley/wheat flour slurries exhibited a higher enzymatic digestion, due to the increased accessibility of starch, boosting the content of reducing sugars [9,10]. Besides changes in matrix composition, HPT alterations of starch are monitored visually by the transformation of starch granules from a spherical/elliptical to an irregular and ruptured shape [9], as well as the loss of birefringence [11,12]. Granular destruction of starch, in turn, increased swelling and initial viscosity when pressures above 500 MPa are applied [12,13].

To predict the extent of high pressure-induced starch modification, the knowledge of influencing factors is indispensable. Kudta and Tomasik observed a pressure and time dependent structural modification of starch [14]. Thereby, HPT of starch in aqueous solution resulted in significant changes of starch, however, (pea) starch suspended in ethanol exhibited no structural and functional changes [13]. Even the addition of salt, as ferric chlorides, to aqueous media affected the modification process [15]. Furthermore, intrinsic factors, such as type of starch (A, B, C) [16], starch composition as amylose/amylopectin ratio [17,18], and the origin of starch [17,19] influence the effectiveness of pressure treatments. 

To date, investigation on the impact of HPT on starch were performed mainly in starch–water slurries [20]. However, during several cereal processing steps, such as grinding, starch is exposed to high pressure without the addition of extra water. Since HPT resulted in a partial replacement of internal starch hydrogen bonds and gelatinization of starch, it is assumed that former exploration cannot be transferred easily to high-pressure treatments on starch or flour in powdered state (≤14%). Insights into the structural alteration of high pressure on starch in cereal matrices, such as flour, are essential to understand starch-modifying processes during grinding and milling. With the aid of highly compressible vials containing the wheat flour sample, HPT could be successfully applied to non-hydrated wheat flour. Thus, the impact of varied pressure (up to 600 MPa) and pressurization time (up to 20 min) on flour characteristics was determined. This study presents the effects of HPT on the structural and functional modification of wheat flour, and on this basis, enables the investigation of HPT on low-moisture products. 

## 2. Materials and Methods 

### 2.1. Material

High pressure was applied to commercial wheat flour (Type 550, variety “Akteur”) provided by Rosenmühle GmBH (Ergolding, Germany). The moisture content of the flour was determined according to the approved method of the AACC 44-40 (2000). Moisture content was constant at 14.64 ± 0.27% before and after treatment. The protein content (10.55 ± 0.03% flours DM (dry matter)) was evaluated using Kjeldahl method AACC 46-12.01 with a conversion factor of 5.7. Ash content was 0.67 ± 0.02 g/100 g analyzed in accordance to AACC 08-12.

### 2.2. High-Pressure Treatment

HPT experiments were performed on a self-constructed high-pressure device at the Chair of Technical Microbiology at Technical University of Munich. Wheat flour was filled in elastic cryotubes, slightly compressed and hermetically sealed. The surrounding housing was filled with polyethylene–glycol–water to achieve a satisfying pressure transfer to the sample tubes. In two experimental test series (Table 1), the impact of pressurization time (at constant pressure) and of pressure (at constant pressurization time) was analyzed. Depending on the applied pressure, pressure build-up varied between 0.75 and 3.00 min (Table 1). The pressurization time started when the applied pressure was reached. All pressure treatments were performed at least in triplicates. 

### 2.3. Molecular Structure of Starch 

Determination of glucose, maltose, maltotriose, as well as fructose and saccharose, was performed using HPAEC-ED (high-performance anion-exchange chromatography with electrochemical detector from Dionex Softron GmbH (Germering, Germany). Flours were dissolved in a methanol–water (1:1) solution (1 g/flour/8 mL solvent) to inhibit enzymatic reactions. Afterwards, samples were filtered through 0.45 μm syringe filters and stored at −18 ± 1 °C before the analysis. Relative amylose content was determined with an amylose/amylopectin assay kit (Megazyme International Ireland Ltd., Wicklow, Ireland). Total starch content was analyzed according to the AACC Methode 76-13.01 using Total Starch Enzyme Kit from Megazyme International Ireland Ltd. (Wicklow, Ireland). To purify starch and remove interfering dextrin of the sample, flour samples were purified in concentrated ethanol solution (80%). 

### 2.4. Crystalline Properties

Impact of HPT on crystalline structures of starch was studied using dynamic scanning calorimetry (DSC) (Pyris Diamond, Perkin Elmer, Waltham, MA, USA) equipped with a cooling system (Intracooler 2P). Flour samples were mixed with distilled water (flour/water = 1:3), filled in aluminum pans (20–35 mg) and hermetically sealed. After 2 min equilibration time at −40 °C, samples were heated from −40 °C to +95 °C, and held again for 2 min. The gelatinization enthalpy (calculated from the peak endotherm) describes the amount of energy which is necessary to melt crystalline parts of starch during heating. 

### 2.5. Granular Structure

Particle size distribution prior and after HPT was analyzed by static light scattering using Mastersizer 3000 equipped with AeroS unit for dry measurements (Malvern Instruments Ltd., Worcestershire, UK). “Mie Theory” was applied to calculate the particle size distribution using a refractive index of 1.59, the “general purpose mode”, and an obscuration ranging between 2 and 8% during measurements. All measurements were performed in triplicates.

### 2.6. Starch Modification Degree

Starch modification degree (formerly known as starch damage) was determined according to the approved method (AACC method 76-31.01) using the Starch Damage Assay Kit (Megazyme International Ireland Ltd., Wicklow, Ireland). The starch modification degree (SMD) specifies the amount of starch which can be hydrolyzed at 40 °C by fungal α-amylase to low molecular weight dextrin, and is calculated using Formula (1):

SMD = ΔE × F × 60 × (1/1000) × (100/W) × (162/180),(1)

with SMD = starch modification degree (%), ΔE = absorbance (reaction) read against the reagent blank, F = (150 μg of glucose)/(absorbance of 150 μg of glucose), W = sample weight (mg).

### 2.7. Hydration Properties

The solvent (water) retention capacity (WRC) of flours states the amount of water which can be bound by the sample after centrifugation at low rotational speed (AACC 56-11.02). The approved method had to be modified due to the low sample amount. The ratio flour/solvent remained constant, however, flour weight was reduced to 1.00 ± 0.05 g, and distilled water to 5.00 ± 0.05 g. 

### 2.8. Statistical Evaluation

The statistical analysis was performed using JMP**^®^** Pro (Version 12.2.0, JMP Software, SAS Institute Inc., Cary, NC, USA). The effects of HPT on starch structures and functionality were determined using Tukey’s pairwise comparisons with a confidence level of 95.0% with ANOVA.

## 3. Results and Discussion

### 3.1. Molecular Alterations of Starch

Starch mainly consists of the highly branched polymer amylopectin and the linear glucan amylose, which determine the functional properties of starch. The polymer constitution of starch in flour samples gives information of the origin of starch and provides fundamental insights into the extent of stress acting on starch during physical treatments. As shown in Table 2, with the increase in pressure from 0 to 600 MPa, a significant rise of relative amylose content from 31.7 ± 1.7% of total starch to 40.3 ± 1.2% of total starch (linearly, *R*^2^ = 0.834), and decrease of total starch from 77.7 ± 0.8% to 67.6 ± 1.7% (linearly, *R*^2^ = 0.892) occurred. The simultaneous increase of percentage amylose content and decrease of total starch can be reduced to the destruction of the starch polymer amylopectin. It is well known from literature, that the degradation of the highly-branched polymer amylopectin (~10^8^ Da in comparison to amylose ~10^6^ Da) is favored when physical forces are applied to pure starch or flours [21,22], explainable by the higher molecular weight and rigidity of amylopectin. The predominant degradation of amylopectin to low molecular weight dextrin or the debranching of this polymer resulted in a percentage rise of amylose in total starch. However, the extent of the degradation of total starch content was surprising. Applied pressure of 600 MPa caused a reduction of the starch content of 13.0%, despite a low moisture content in the present flour samples (14.64 ± 0.27%). Extrusion processes of starchy matrices are known to reduce, extremely, the hydrodynamic radius of starches caused by the splitting of polymer chains, as well [23]. However, this study was performed in matrices with glycerol/water plasticizer of 30 or 40%, facilitating cleavage of polymer chains. 

Although the determination of total starch content using a Megazyme assay kit presents a fast and reproductive method, it is questionable if an underestimation of the dextrin content after severe physical treatment of starchy matrices occurs. In the first step of this assay, interfering substances are removed by washing the sample in ethanol. The cleavage of starch leads to the formation of low molecular weight fragments showing a higher solubility than starch. Those dextrins are removed prior to the enzymatic digestion, and thus, not detected. 

A slight (significant) decrease of maltose content was further investigated, when pressure increased from 0 to 600 MPa (0.8 ± 0.1 to 0.6 ± 0.1 mg/g flour). To date, it remains unclear if the reduction of maltose content is due to a mechanically induced splitting of the disaccharide. An enzymatic hydrolyzation of starch was excluded, since enzymatic degradation of maltose would cause an increase in glucose content, which could not be determined so far (data not shown), and furthermore, temperature did not rise during the pressure treatment above (30 ± 1 °C). Although decrease in maltose content is negligible for practical application, it provides insights into the mechanism of high pressure on the molecular modification of starch. 

Surprisingly, decrease in maltose content could not be determined with an increase in pressurization time from 0 to 20 min at a pressure level of 450 MPa. Furthermore, the analysis of the impact of pressurization time on starch structures elucidates the time-dependent destruction of starch. Due to the natural logarithmic destructive behavior of starch with increase in pressurization time, during the first 5 min of the treatment, the total starch content decreased about 5.1%; within the following 15 min (min 5–20), starch content only decreased about 5.8%. 

Due to the huge impact of HPT, even in low moisture matrices on starch, pressure treatments of food systems should not be neglected. Since a favored destruction of amylopectin occurred during HPT, measurements of starch crystallinity are necessary to predict the modified behavior of HPT starches during non-isothermal processes.

### 3.2. Alterations in Gelatinization Properties and Starch Modification Degree of Flours 

**Figure 1:**
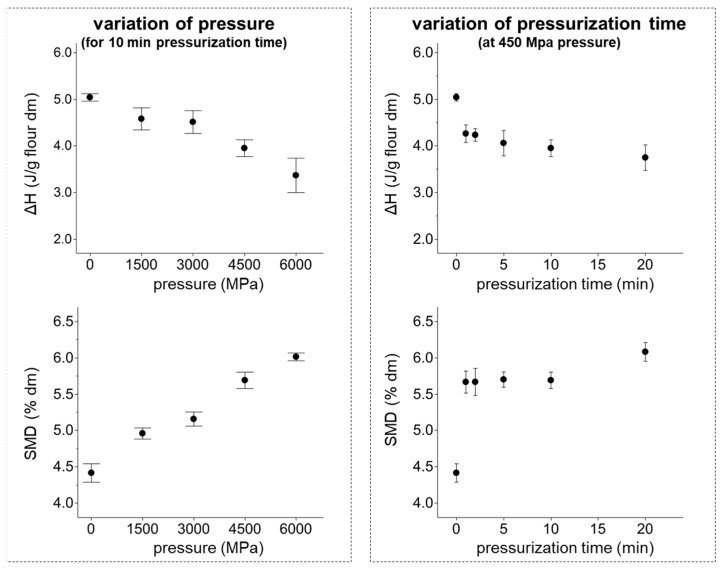
Impact of pressure and pressurization time on the gelatinization enthalpy (ΔH) and starch modification degree (SMD) of wheat flour, $\bar{x}$ ± SD, *n* = 3.

Alterations in starch gelatinization properties on nanoscopic scale can be visualized using differential scanning calorimetry. In starch–water suspensions, a destruction of starch crystallinity was already determined by differential scanning calorimetry (DSC) and X-ray method [24]. These results coincide with findings in these studies, despite the significant lower moisture content in the present study. With increase in pressure, a linear decrease in the gelatinization enthalpy of HPT samples from 5.0 ± 0.1 J/g to 3.4 ± 0.4 J/g was noticed (*R*^2^ = 0.851) (Figure 1). Thus, the pressure treatment of 600 MPa resulted in a reduction of total starch and crystallinity of 13.0 and 32.6%, respectively. Consequently, reduction of the total starch content could entail the multiple decline of crystallinity. Furthermore, the severe reduction of gelatinization enthalpy could be referred to a partial gelatinization of starch. In high-moisture matrices, 15–88% of wheat starch was melted, when pressure between 300 and 500 MPa was applied [25]. Thus, a partial gelatinization of starch in HPT non-hydrated samples is possible, causing an accelerated decline in gelatinization enthalpy. The stepwise increase in pressurization time up to 20 min caused a reduction of gelatinization enthalpy to 3.8 ± 0.3 J/g, following a natural logarithmic curve shape (comparable to total starch content): within the first minute of treatment (plus pressure built-up time), gelatinization enthalpy decreased to 4.3 ± 0.2 J/g. Afterward, a decelerated decline in gelatinization enthalpy was noticed, which is consistent with findings of Bauer and Knorr [26]. In general, severe alterations of starch crystallinity were preceded by destruction of granular constitution of starch, and thus facilitated enzymatic digestion.

The starch modification degree (SMD) increased with pressure level (up to 600 MPa) by 37% (linear dependency, *R*^2^ = 0.97). A dependency of SMD from the applied pressure was also revealed by Ahmed et al. on matrices containing 20–50% wheat flour [27]. The rise in pressurization time (up to 20 min) resulted in an increase of SMD of 38%. At the applied pressure of 450 MPa, main alterations in SMD occurred during the first minute of treatment. Thereby, SMD was raised from 4.40 ± 0.13% to 5.66 ± 0.15%, and stayed constant during the following 9 min, demonstrating the need for a detailed control of HPT-induced alterations of starchy matrices, especially during short HPT.

Thereby, the starch modification degree of flours is a key value for the enzymatic degradation of flour particles by amylases during fermentation processes. To draw conclusions regarding the enzymatic hydrolysis and as well to monitor physically-induced changes of flours, the particle size distribution of flours is often determined. Furthermore, changes on microscopic scale can be identified using static light scattering.

### 3.3. Granular Modification of Wheat Flour Particles

Wheat flours, modified by grinding procedures, showed in previous studies a good correlation of SMD and the particle size distribution, so that authors assumed that rise in SMD was caused by the surface enlargement due to the particle size reduction [22]. However, for HPT flours, no reduction of bigger flour particles was visible, as demonstrated by the D_3,90_ of the pressure and pressurization time test series (Table 3). Low pressurization time of 1 min caused a significant reduction of the medium particle size D_3,50_ from 90.4 ± 0.8 to 74.0 ± 4.7 µm without any alterations for longer treatments. A comparable behavior was detected for the pressure level, whereby for pressure treatments above 150 MPa, no significant changes of the medium particle size D_3,50_ were noticed anymore. In literature, contradictory results and dependencies concerning particle size alterations of HPT starches were found: while application of high pressure caused a disruption of bigger particles in high moisture wheat slurry (>50%) [27], an increase of particle size of starch by five- to six-fold was noticed in almond milk or wheat slurries containing sugars [28,29]. However, in the present study, no alterations of flour particles were visible for low moisture matrixes as non-hydrated wheat flour (14%), which is in accordance to the findings of Douzals on wheat starch suspensions (5% DM) [30].

Since particle size distribution (PSD) values are in contrast with the development of SMD under pressure treatments in non-hydrated matrices, where a continuous increase in SMD over the whole pressure range was measured, PSD is not, in general, an adequate analytical method for the prediction of the accessibility of particles for enzymatic digestion. Consequently, surface alterations of wheat flour particles occur, which is not related to the particle size reduction. Changes of starch–gluten agglomerates are known to occur by mechanical treatments, for example grinding, even in low moisture matrices, due to the removal of starch-covering proteins by friction forces. However, since during HPT, 3-dimensional and low friction forces are applied on starch–protein agglomerates, no removal of starch-covering proteins was expected, increasing the accessibility of starch. Therefore, possible explanations for the rise in SMD are microcracks and the deformation of starch granules, which enlarge the surface of starch, and consequently, enzymatic accessibility, without altering noticeably the particle size distribution.

To summarize, high pressure-treated flours show comparable molecular (total starch content), crystalline (gelatinization enthalpy) and surface-related (enzymatic accessibility) alterations, which cannot be correlated to the modification of particle size. Thus, using static light scattering, HPT-caused alterations of wheat flours cannot be determined. For the application of pressure-treated starchy matrices in food industry, beside structural alterations, the functional properties of HPT flours are of special interest, too.

### 3.4. Facilitated Hydration of HPT Flours

**Figure 2:**
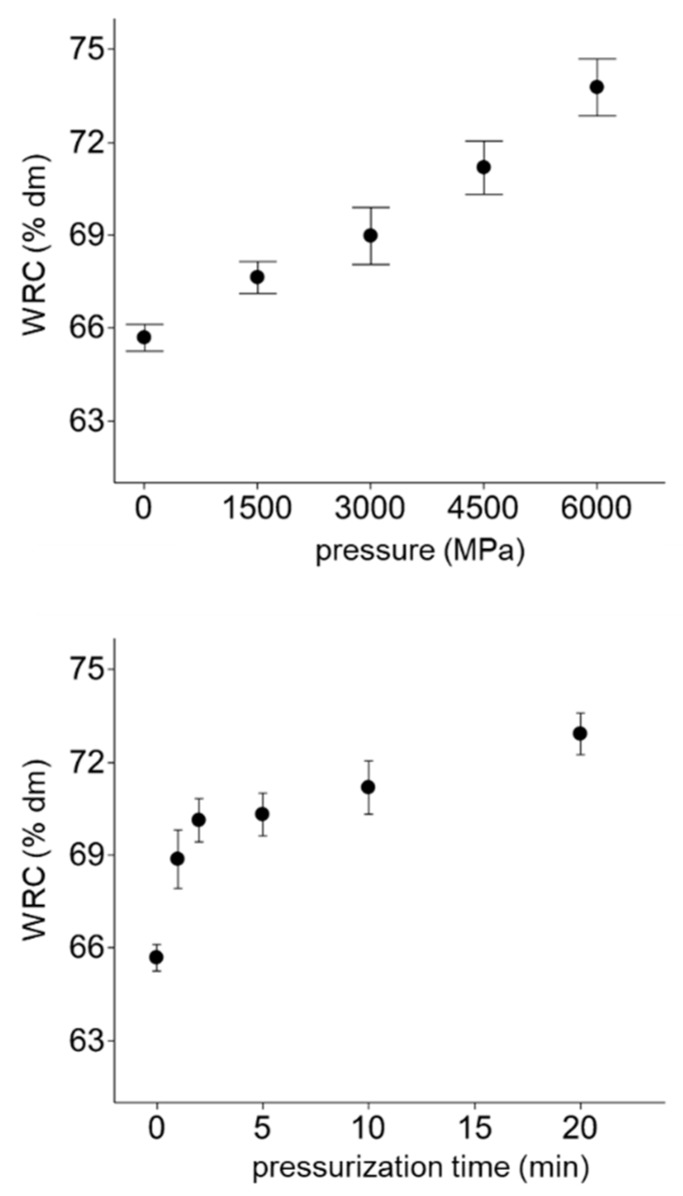
Impact of pressure and pressurization time on hydration properties (WRC = water retention capacity) of wheat flour, $\bar{x}$ ± SD, *n* = 3.

The water retention capacity (WRC) of flours describes the amount of water which can be bound by flours after centrifugation. For an increase in WRC, a high accessibility of flours to water is prerequisite. Existing studies in high-moisture matrices have already shown a dependency of pressure and the swelling index of wheat starch [31], and a higher retention capacity of medium moisture matrices (~40%) treated by pressure treatment at 600 MPa [32]. Those findings are also mainly transferable to non-hydrated, starch-based flours. Both test series (pressure level and pressurization time) led to a significant increase in WRC, according to the curve shapes of SMD (compared in Figure 2). The increase in pressure up to 600 MPa resulted in a linear rise of WRC (*R*^2^ = 0.942) from 65.68 ± 0.43% to 73.77 ± 0.92%. This is in agreement with findings from authors dealing with alterations of flour hydration introduced by high-pressure treatments in starch suspensions. However, less effects of high-pressure on starch or flour are expected, as in comparison to HPT of starch suspension, since the hydration highly depends on the availability of free water [33]. A pressurization time of 2 min at 450 MPa caused an increase of WRC to 70.12 ± 0.70%, however, afterwards, only a slight increase of WRC was noticed (72.91 ± 0.67% for 20 min HPT). The relation of starch modification degree, pressure, and increased hydration properties of starch in dispersions was also noticed by Ahmed et al. [27]. Hence, the hypothesis can be confirmed, that high-pressure treatment of low moisture starchy matrices significantly modifies the flour particle accessibility, as demonstrated by SMD and WRC measurements, without evoking severe alterations of particle size.

## 4. Conclusions

High-pressure treatment of non-hydrated starch–protein particles causes structural modifications of starch, and consequently, functionality of the matrix. Thus, evaluation of high-pressure treatments of starchy food should not exclusively focus on the improvement of the microbiological stability of food products, but also consider functional alterations of the matrix by HPT. These findings are of special interest for the practical, industrial application. The reduction of microbiological stability, in combination with a better enzymatic accessibility of starch, facilitates long-term fermentation processes under controlled conditions. Since high-pressure treatments of starchy matrices, even under low moisture conditions, evoke modifications of starch, grinding processes with (proportional) compressive forces—as found in roller grinding processes—should be examined and adjusted precisely, to prevent undesired alterations. This knowledge can lead to a reconsideration of high-pressure treatments of food systems, and to a targeted selection of monitoring parameters during HPT.

## Figures and Tables

**Table 1:** High-pressure treatment (HPT) parameters applied to wheat flour sample and pressure built-up time.

Pressure (MPa)	Pressurization Time (min)	Pressure Build-Up (min)
0	10	0
150	10	0.75
300	10	1.50
450	10	2.25
600	10	3.00
450	1	2.25
450	2	2.25
450	5	2.25
450	10	2.25
450	20	2.25

**Table 2:** Molecular modification of starch in wheat flour visualized by the maltose content, amylose content, and total starch content in dependency of pressurization time and pressure, $\bar{x}$ ± SD, *n* = 3, DM: dry matter.

Variation of Pressure (for 10 min Pressurization Time)				Variation of Pressurization Time (at 450 MPa)			
Pressure (MPa)	Maltose (mg/g Flour)	Amylose (% of Total Starch)	Total Starch (% DM)	Pressurization Time (min)	Maltose (mg/g Flour)	Amylose (% of Total Starch)	Total Starch (% DM)
0	0.8 ± 0.1 ^A^	31.7 ± 1.7 ^C^	77.7 ± 0.8 ^A^	0	0.8 ± 0.1 ^A^	31.7 ± 1.7 ^C^	77.7 ± 0.8 ^A^
150	0.8 ± 0.1 ^A,B^	33.2 ± 1.2 ^B,C^	76.8 ± 0.8 ^A,B^	1	0.7 ± 0.1 ^A^	34.2 ± 1.0 ^B,C^	76.2 ± 0.9 ^A,B^
300	0.6 ± 0.1 ^B^	35.2 ± 0.5 ^B,C^	74.0 ± 0.5 ^B,C^	2	0.6 ± 0.1 ^A^	35.7 ± 0.7 ^B^	74.7 ± 0.5 ^B,C^
450	0.6 ± 0.0 ^B^	37.0 ± 1.3 ^A,B^	71.7 ± 0.7 ^C^	5	0.7 ± 0.2 ^A^	36.2 ± 0.6 ^B^	73.7 ± 0.3 ^C,D^
600	0.6 ± 0.1 ^B^	40.3 ± 1.2 ^A^	67.6 ± 1.7 ^D^	10	0.6 ± 0.0 ^A^	37.0 ± 1.3 ^A,B^	71.7 ± 0.7 ^D^
				20	0.6 ± 0.1 ^A^	40.4 ± 0.7 ^A^	69.4 ± 0.4 ^E^

Different letters in columns mark statistically significant differences between means (*p* ≤ 0.05).

**Table 3:** Granular alterations of wheat flour particles determined by the particle size distribution (volume) in dependency of pressurization time and pressure, $\bar{x}$ ± SD, *n* = 3.

Variation of Pressure (10 min Pressurization Time)				Variation of Pressurization Time (450 MPa)			
Pressure (MPa)	D_3,10_ (µm)	D_3,50_ (µm)	D_3,90_ (µm)	Pressurization Time (min)	D_3,10_ (µm)	D_3,50_ (µm)	D_3,90_ (µm)
0	24.2 ± 0.4 ^A^	90.4 ± 0.8 ^A^	185.2 ± 5.9 ^A^	0	24.2 ± 0.4 ^A^	90.4 ± 0.8 ^A^	185.2 ± 5.9 ^A^
150	19.8 ± 0.4 ^A^	81.5 ± 1.7 ^B^	184.5 ± 7.3 ^B^	1	17.6 ± 1.1 ^C^	74.0 ± 4.7 ^B^	182.8 ± 4.5 ^A,B,C^
300	19.9 ± 0.6 ^A^	82.2 ± 1.5 ^B^	184.4 ± 2.3 ^B^	2	17.4 ± 1.3 ^C^	73.5 ± 5.1 ^B^	179.0 ± 3.3 ^B,C^
450	20.2 ± 2.1 ^A^	81.0 ± 6.5 ^B^	184.3 ± 6.1 ^B^	5	18.0 ± 1.4 ^C^	75.9 ± 5.2 ^B^	177.9 ± 3.0 ^C^
600	21.1 ± 2.0 ^A^	83.4 ± 7.1 ^B^	191.7 ± 9.9 ^B^	10	20.2 ± 2.1 ^B^	77.1 ± 4.9 ^B^	184.3 ± 6.1 ^A,B^
				20	17.0 ± 0.9 ^C^	71.7 ± 3.8 ^B^	179.6 ± 3.7 ^A,B,C^

Different letters in columns mark statistically significant differences between means (*p* ≤ 0.05).
